# Predictive effect of TCED-HFV grading and imaging biomarkers on anti-VEGF therapy in diabetic macular edema

**DOI:** 10.1186/s12886-023-02973-7

**Published:** 2023-05-23

**Authors:** Lu Yu, Xiaolin Hao, Jie Cheng, Yu Ling, Hong Ren, Bin Mo, Wu Liu

**Affiliations:** 1 Department of Ophthalmology, Beijing Tongren Hospital, Beijing Tongren Eye Center, Capital Medical University, 1 Dongjiaominxiang Road, Beijing, 100073 P.R. China; 2 Beijing Ophthalmology and Visual Sciences Key Laboratory, Capital Medical University, Beijing, 100073 P.R. China; 3 Department of Ophthalmology, Aerospace Center Hospital, Peking University Aerospace School of Clinical Medicine, Beijing, 100049 P.R. China

**Keywords:** Diabetic macular edema, Optical coherence tomography, Anti-VEGF

## Abstract

**Background:**

To evaluate the predictive effect of TCED-HFV grading and imaging biomarkers on anti-vascular endothelial growth factor (anti-VEGF) treatment in diabetic macular edema (DME).

**Methods:**

81 eyes of 81 DME patients who were treated with anti-VEGF were included in this retrospective cohort study. All patients underwent a comprehensive ophthalmic examination at baseline and follow-up, including best-corrected visual acuity (BCVA), fundus photography, and spectral domain–optical coherence tomography (SD-OCT). Baseline imaging biomarkers were qualitatively and quantitatively graded according to the TCED-HFV classification protocol, and DME was divided into early stage, advanced stage, severe stage, and atrophy stage.

**Results:**

Six months post treatment, central subfield thickness (CST) in 49 eyes (60.5%) had decreased by 10% from baseline, 30 eyes (37.0%) had achieved CST < 300 μm, and 45 eyes (55.6%) had BCVA improved by more than five letters. Multivariate regression analysis revealed that eyes with baseline CST ≥ 390 μm had a higher probability of ≥ 10% reduction in CST from baseline, and eyes with abundant hyperreflective dots (HRD) had a lower probability of 10% reduction in CST (all *P* < 0.05). Eyes with vitreomacular traction (VMT) or epiretinal membrane (ERM) at baseline were less likely to reach the end point of CST < 300 μm (*P* < 0.05). BCVA increases of more than five letters were less likely in eyes with baseline BCVA ≥ 69 letters, complete or partial destruction of ellipsoid zone (EZ) at baseline (all *P* < 0.05). TCED-HFV staging was negatively correlated with BCVA at both baseline and 6 months (Kendall’s tau-b=-0.39 and − 0.55, all *P* < 0.01). TCED-HFV staging was positively correlated with CST at 6 months (Kendall’s tau-b = 0.19, *P* = 0.049) and negatively correlated with the reduction of CST (Kendall’s tau-b=-0.32, P < 0.01).

**Conclusion:**

The TCED-HFV grading protocol facilitates a comprehensive assessment of DME severity, standardizes the grading of multiple imaging biomarkers, and predicts the anatomical and functional outcomes of anti-VEGF treatment.

**Supplementary Information:**

The online version contains supplementary material available at 10.1186/s12886-023-02973-7.

## Background

Over the past few decades, the prevalence of diabetes has increased throughout the world, including in China. A population-based cross-sectional study revealed that the prevalence of adult diabetes in China was 12.8%, with an estimated total of 129.8 million Chinese mainland diabetes patients [1]. Among the main complications of diabetes, diabetic retinopathy (DR) and diabetic macular edema (DME) are the leading causes of visual impairment in working-age people. In a study of Handan Ophthalmology in northern China, the prevalence of DME in people over 40 years old with diabetes was 5.2% [2], and it was conservatively estimated at least 6.75 million DME patients in China [1, 2].

Anti-vascular endothelial growth factor (anti-VEGF) is widely used to treat DME and improve vision outcomes [3, 4]. Although approximately 50% of visually impaired patients in standard clinical trials experienced an improvement in two-line visual acuity (VA) on the ETDRS visual acuity chart within two years, however, patient responses to VEGF treatment varied, with some patients responding poorly to VEGF [5, 6]. A posthoc analysis revealed that nearly 40% of patients who received 3 months of anti-VEGF therapy had an improvement in best-corrected visual acuity (BCVA) of less than five letters [7]. The reality is more complex, and predicting the effect of anti-VEGF therapy is a common concern of patients and clinicians.

The detailed features of spectral domain–optical coherence tomography (SD-OCT) and color fundus images can reflect the severity of DME and predict the prognosis of patients. However, because of many relevant biomarker descriptions, it is challenging to perform comprehensive analysis in clinical applications. In 2020, the International Retinal Expert Group of the European Institute for Advanced Studies in the Classification of Ophthalmology (ESASO) created a classification of diabetic macular disease (DM) based solely on SD-OCT, with reference to the specific morphological characteristics and quantitative indicators from previous OCT studies on DME [8], A “TCED-HFV” grading system including seven qualitative and quantitative indicators was proposed, which may be helpful for comprehensive assessment and a personalized follow-up of DME patients in clinical practice [9]. The present study evaluated the predictive effect of the aforementioned classification system for anti-VEGF treatment in DME patients and assessed the relative importance of these factors.

## Methods

We retrospectively reviewed the medical records of patients who received more than 3 times of continuous anti-VEGF treatment for DME in the Department of Ophthalmology at the Aerospace Central Hospital from January 2018 to January 2022 and were followed up for more than 6 months. Inclusion criteria: age > 18 years old; diagnosed with type 2 diabetes mellitus; the presence of DR-related DME. Diagnosis of DME was based on SD-OCT (Spectralis HRA + OCT; Heidelberg Engineering, Heidelberg, Germany) measured by built-in software with a central thickness of more than 300 μm within 1 mm. The study followed the principles of the Helsinki Declaration, and the Institutional Review Committee of The Aerospace Central Hospital approved the study and waived the requirement for informed consent in this retrospective study.

Exclusion criteria include a history of uveitis, patients with high myopia (diopter less than − 6D), glaucoma, other retinal disorders (e.g., RVO, macular hole, age-related macular degeneration, or angioid streaks), refractive interstitial opacity that cannot obtain clear image quality because of any cause (e.g., cataract, vitreous hemorrhage, or corneal disease), patients with a history of vitrectomy, had undergone intraocular surgery or intraocular laser therapy 6 months before treatment. Patients who received treatment for DME other than anti-VEGF therapy and panretinal photocoagulation (PRP) during the subsequent 6-month follow-up period were also excluded. None of the patients received macular grid photocoagulation before treatment and during follow-up. If both eyes of a patient met the inclusion criteria, one eye was randomly selected.

Basic information includes gender, age, course of diabetes, renal function, hypertension, and cardiovascular and cerebrovascular diseases. If measured within 3 months before baseline, the glycosylated hemoglobin (HbA1c) level was recorded. The ophthalmological examination included intraocular pressure (IOP), slit lamp biomicroscope, color fundus photos, and SD-OCT images. BCVA was measured using Snellen charts. According to the previously described method, the Snellen VA measurements were converted to approximate ETDRS study letter scores for statistical operations [10].

DME and Imaging Biomarkers Grading: According to the Early Treatment of Diabetic Retinopathy Study (ETDRS), color fundus photographs were used to determine whether the affected eye underwent PRP and graded the hard exudation (HE) state [11]. Qualitative and quantitative OCT parameters of tomography were graded according to the DME grading protocol described by the “TCED-HFV” grading system [8], including CST, intraretinal cysts (IRC), subretinal fluid (SRF), the ellipsoid zone (EZ) status, disorganization of the inner retinal layers (DRIL), hyperreflective dots (HRD), and vitreoretinal relationship. According to the first four parameters (CST, IRC, EZ, and DRIL), DME was divided into early, advanced, severe, and atrophic stages.

Biomarkers were scored independently by two experienced ophthalmologists (H.XL. and Y.L.). For each biomarker, if there were a difference in the evaluation between the two raters, a third rater (C.J.) would be convened to reach a consensus.

For statistical analysis, the normality of variables was tested using the Shapiro-Wilk W test. Classified and continuous variables are expressed as No. (%) and mean ± standard deviation. The changes in BCVA and CST at baseline and 6-month follow-up were compared using the paired sample *t*-test. Univariate Logistics regression analysis was used to analyze the potential correlation between the basic data and ophthalmic imaging features and the results of a decrease in CST greater than 10% from baseline, CST decreased to less than 300 μm and BCVA improved ≥ 5 ETDRS letters at the end point of follow-up (6 months). The variables with *P* ≤ 0.2 in the univariate Logistics regression model were selected for multivariate Logistics regression analysis, and the variance expansion factor (VIF) was checked to evaluate the multi-collinearity among the variables. The chi-square test was used for inter-group comparison, and Kendall’s tau-b test was used for correlation analyses. Multiple comparisons were performed using Kruskal-Wallis H tests. *P* < 0.05 was statistically significant. All data were analyzed using *SPSS for Windows*, *Version* 24.0. *Chicago, IL: SPSS Inc.*

## Results

Table 1 presents the demographic and baseline characteristics of the study population, and the study included 81 eyes from 81 patients aged 58.0 ± 9.0 years with a mean HbA1c of 7.9 ± 2.3%. Fifteen eyes (18.1%) had received PRP.

**Fig. 1:**
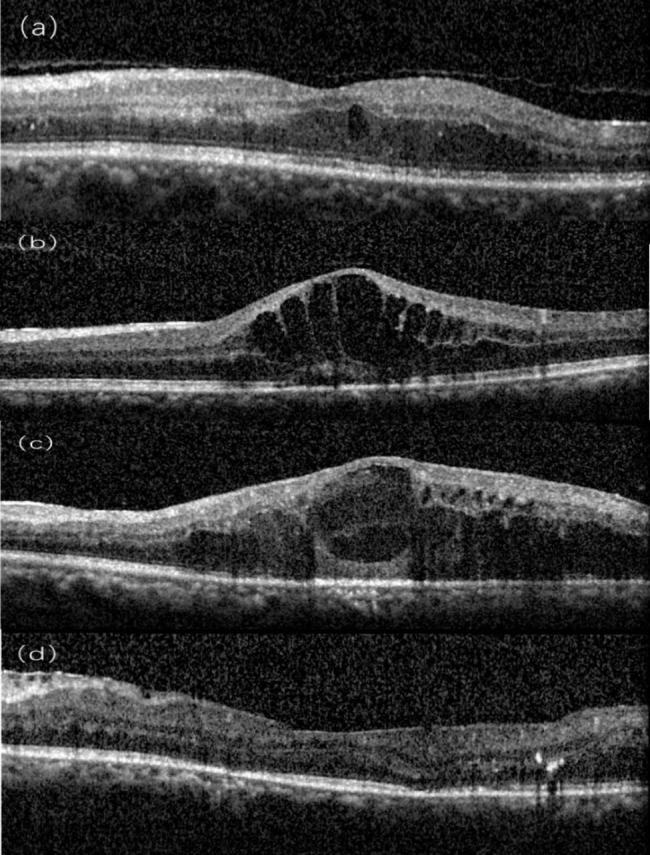
SD-OCT Staging of diabetic maculopathy (a) Early diabetic macular edema. Multiple small cystoid spaces in the outer nuclear layer, with mild thickening of the macula. Incomplete vitreous detachment. Hyperreflective foci are more than 30 in number. The TCED-HFV grading is T = 1; C = 1; E = 0; D = 0; H = 1; F = 0; V = 1. (b) Advanced diabetic macular edema. Intermediate cystoid spaces in the macula. The ellipsoid zone is not gradable but the external limiting membrane is disrupted subfoveally. The segmentation of the internal retinal layers is still visible. Absence of any visible adhesion or traction is discernible. The TCED-HFV grading is T = 2; C = 2; E = 1; D = 0 H = 0; F = 1; V = 0 (c) Severe diabetic macular edema. Central macrocyst surrounded by cystoid spaces involving the outer nuclear layer, the outer plexiform layer, and the inner nuclear layer. The external liming membrane and the ellipsoid zone are not discernible subfoveally. Retinal inner layers are damaged but still visible (no DRIL), while vitreoretinal relationship is normal. The TCED-HFV grading is T = 2; C = 3; E = 2; D = 0; H = 0; F = 0; V = 0.(d) Atrophic diabetic maculopathy. Central retinal thinning with disorganization of the inner retinal layers (DRIL) and epiretinal membrane. The external liming membrane and the ellipsoid zone are not discernible subfoveally, and the retinal pigment epithelium is partly atrophic. The TCED-HFV grading is T = 0; C = 1; E = 2; D = 1; H = 1; F = 0; V = 4

Table 1 lists baseline image biomarkers. 41 eyes (50.6%) had CST greater than 390 μm, 19 eyes (23.5%) had severe IRC, 45 eyes (55.6%) had destruction or absence of the EZ, and DRIL was present in 40 eyes (49.4%), 19 eyes (23.5%) had SRF in the fovea, HRD was graded as abundant (greater than 30 points) in 48 eyes (59.3%). 20 eyes (24.7%) had epiretinal membranes (EMR). Combining CST, IRC, EZ, and DRIL states, DME stages were obtained: early (16%), advanced (55.6%), severe (18.5%), and atrophy (9.9%) (Fig. 1). Fundus image revealed moderate to severe HE in 22 eyes (27.2%) at baseline.

**Table 1:** Demographic and baseline characteristics

Characteristics	Baseline
No. of patients	81
No. of eyes	81
Age, mean ± SD, y	58.0 ± 9.0
Male, No. (%)	48(59.3)
Under renal dialysis, No. (%)	1(1.2)
Previous hypertension, cardiovascular and cerebrovascular disease, No. (%)	45(55.6)
HbA1c (%), mean ± SD	7.9 ± 2.3
Diabetes mellitus duration, mean ± SD, y	13.2 ± 7.7
Prior PRP, No. (%)	15(18.1)
**Diabetes retinopathy grading, No. (%)**	
Mild or moderate NPDR	6(7.4)
Severe or very severe NPDR	30(37.0)
PDR	21(25.9)
High-risk PDR	9(11.1)
Cannot grade^a^	15(18.1)
Lens status, phakic, No. (%)	46(56.8)
**DME imaging biomarkers**	
**Central subfield foveal thickness, No. (%)**	
300–329 μm	8(9.9)
330–389 μm	18(22.2)
≥ 390 μm	55(67.9)
**Intraretinal cysts, No. (%)**	
Absent	0
Mild	38(46.9)
Moderate	24(29.6)
Severe	19(23.5)
**Ellipsoid zone status, No. (%)**	
Intact	36(44.4)
Disrupted	22(27.2)
Absent	23(28.4)
**Presence of DRIL, No. (%)**	40(49.4)
**Presence of subretinal fluid, No. (%)**	19(23.5)
**Hyperreflective dots, No. (%)**	
Absent or scarce (< 30)	33(40.7)
Abundant (≥ 30)	48(59.3)
**Foveal Hard exudates, No. (%)**	
Absent	33(40.7)
Mild	26(32.1)
Moderate	17(21.0)
Severe	5(6.2)
**Vitreoretinal relationship, No. (%)**	
Complete posterior vitreous detachment	16(19.8)
incomplete posterior vitreous detachment	29(35.8)
Vitreomacular traction	16(19.8)
Epiretinal membrane	20(24.7)
**DME stage**^**b**^, **No. (%)**	
Early DME	13(16.0)
Advanced DME	45(55.6)
Severe DME	15(18.5)
Atrophic maculopathy	8(9.9)

^a^Eyes with prior PRP were non-gradable. ^b^DME stage was graded according to Panozzo et al.’s [7] study

Anatomical and functional results after 6 months: At 6 months, the average number of injections was 4.27 ± 0.87. BCVA ETDRS score increased from 56.2 ± 18.2 to 61.0 ± 15.9. The mean ETDRS letters gained was 4.81 ± 13.1; the difference was statistically significant (*P* < 0.01). BCVA improved by greater than or equal to five letters in 45 eyes (55.6%), remained stable in 24 eyes (29.6%), and decreased vision by more than five letters in 12 eyes (14.8%). Anatomical examination revealed that CST decreased from 440.6 ± 99.8 μm at baseline to 318.3 ± 49.5 μm (*P* < 0.01) after 6 months, of which 49 eyes (60.5%) had CST decrease by ≥ 10% from baseline, 30 eyes (37.0%) reached CST < 300 μm after 6 months.

Univariate analysis of biomarkers associated with anti-VEGF treatment outcomes: To simplify the analysis, we combined the grading of some biomarkers, including IRC, EZ, HE, and vitreoretinal relationship. The univariate Logistic regression analysis was then performed (Table 2).

**Table 2:** Univariate analysis for predictors of 6-month outcomes in diabetic macular edema treated with anti-VEGF

Variables	Endpoint 1: CST decreased ≥ 10% from baseline			Endpoint 2: CST decreased to < 300 μm			Endpoint 3: BCVA improved ≥ 5 ETDRS letters		
	Beta	OR (95% *CI*)	*p*-value	Beta	OR (95% *CI*)	*p*-value	Beta	OR (95% *CI*)	*p*-value
Age	-0.03	0.97(0.93–1.03)	0.31	-0.00	1.0(0.94–1.04)	0.74	0.00	1.00(0.95–1.05)	0.95
Male	-0.01	0.99(0.40–2.46)	0.99	-0.61	0.55(0.22–1.37)	0.20	0.28	1.32(0.54–3.21)	0.54
HbA1c	0.12	1.13(0.92–1.38)	0.25	0.00	1.00(0.82–1.23)	0.98	0.07	1.07(0.88–1.30)	0.49
Prior PRP	0.71	2.03(0.58–7.03)	0.36	0.49	1.64(0.53–5.08)	0.40	0.22	1.25(0.40–3.91)	0.70
Phakic lens status	0.67	1.95(0.79–4.83)	0.15	0.43	1.54(0.61–3.87)	0.36	0.50	1.65(0.68–4.01)	0.27
Anti-VEGF 3 times	Reference			Reference			Reference		
Anti-VEGF 4 times	0.22	0.80(0.21–3.03)	0.74	0.81	2.25(0.49–10.34)	0.30	0.51	1.67(0.43–6.51)	0.46
Anti-VEGF 5 times	1.28	3.60(1.22–10.62)	0.02^*^	1.41	3.11(1.20–9.12)	0.03*	0.09	0.91(0.33–2.55)	0.86
Baseline BCVA ≥ 69 letters	-0.03	0.97(0.35–2.73)	0.96	0.17	1.18(0.42–3.33)	0.75	-1.12	0.33(0.11–0.94)	0.04^*^
CST ≥ 390 μm	1.62	3.04(1.85–3.73)	0.01^*^	0.41	1.50(0.56–4.05)	0.42	0.79	2.21(0.85–5.71)	0.10
Severe IRC (reference: moderate or less IRC)	0.23	1.25(0.41–3.82)	0.69	-1.76	0.17(0.04–0.81)	0.03*	-0.43	0.65(0.22–1.90)	0.43
Disrupted or absent EZ (reference: intact EZ)	-0.91	0. 40(0.16–1.02)	0.06	-0.36	0.70(0.28-1,73)	0.44	-2.30	0.10(0.03–0.29)	< 0.01^**^
DRIL present	-0.88	0.41(0.16–1.03)	0.06	-0.82	0.44(0.17–1.11)	0.08	0.16	1.17(0.49–2.81)	0.73
Abundant HRD	-1.67	0.19(0.07–0.54)	< 0.01^**^	-0.83	0.44(0.17–1.10)	0.08	-0.56	0.57(0.23–1.42)	0.23
Subretinal fluid present	0.45	1.57(0.53–4.66)	0.42	0.28	1.32(0.46–3.77)	0.60	0.41	1.51(0.52–4.34)	0.45
Moderate or severe HE (reference: absent or mild HE)	-0.08	0.92(0.34–2.50)	0.88	0.31	0.73(0.26–2.06)	0.55	-0.31	0.74(0.28–1.96)	0.54
Presence of VMT and ERM (reference: PVD and IVD)	-1.24	0.29(0.12–0.74)	0.01^*^	-1.74	0.18(0.06–0.50)	< 0.01^**^	-1.03	0.38(0.14–0.89)	0.03^*^
DME stage ^a^			0.04^*^			0.35			0.13
Early DME	Reference			Reference			Reference		
Advanced DME	-0.80	0.45(0.09–2.30)	0.34	-0.56	0.57(0.17–1.98)	0.38	-0.41	0.67(0.18–2.50)	0.55
Severe DME	-2.11	0.12(0.02–0.75)	0.02^*^	-1.17	0.31(0.06–1.51)	0.15	-1.50	0.22(0.05–1.09)	0.06

*P < 0.05; **P < 0.01. ^a^ DME stage was graded according to a previous study [7]

More than or equal to a 10% reduction in CST from baseline values at 6 months was associated with five intravitreal injections (*OR* 3.6; 95% *CI* 1.22–10.62; *P* = 0.02) and baseline CST ≥ 390 μm (*OR* 3.04; 95% *CI* 1.85–3.73; *P* = 0.01). Meanwhile, abundant HRD (*OR* 0.19; 95% *CI* 0.07 ~ 0.54; *P* < 0.01), presence of VMT or ERM at baseline (*OR* 0.29; 95% *CI* 0.12–0.74; *P* = 0.01), and severe DME (*OR* 0.12; 95% *CI* 0.02–0.75; *P* = 0.02) had negative effects on the CST decreased ≥ 10%. At the end of follow-up, CST decreased to less than 300 μm was associated with five intravitreal injections (*OR* 3.11; 95%*CI* 1.20–9.12; *P* = 0.03), Severe IRC at baseline (*OR* 0.17; 95% *CI* 0.04–0.81; *P* = 0.03) and VMT or ERM at baseline (*OR* 0.18; 95% *CI* 0.06–0.50; *P* < 0.01). Negative predictors of BCVA improvement by more than five letters included baseline BCVA ≥ 69 letters (*OR* 0.33; 95% *CI* 0.11–0.94; *P* = 0.04), presence of VMT or ERM at baseline (*OR* 0.38; 95% *CI* 0.14–0.89; *P* = 0.03), and complete disappearance or partial destruction of EZ (*OR* 0.10; 95% *CI* 0.03–0.29; *P* < 0.01).

**Fig. 2:**
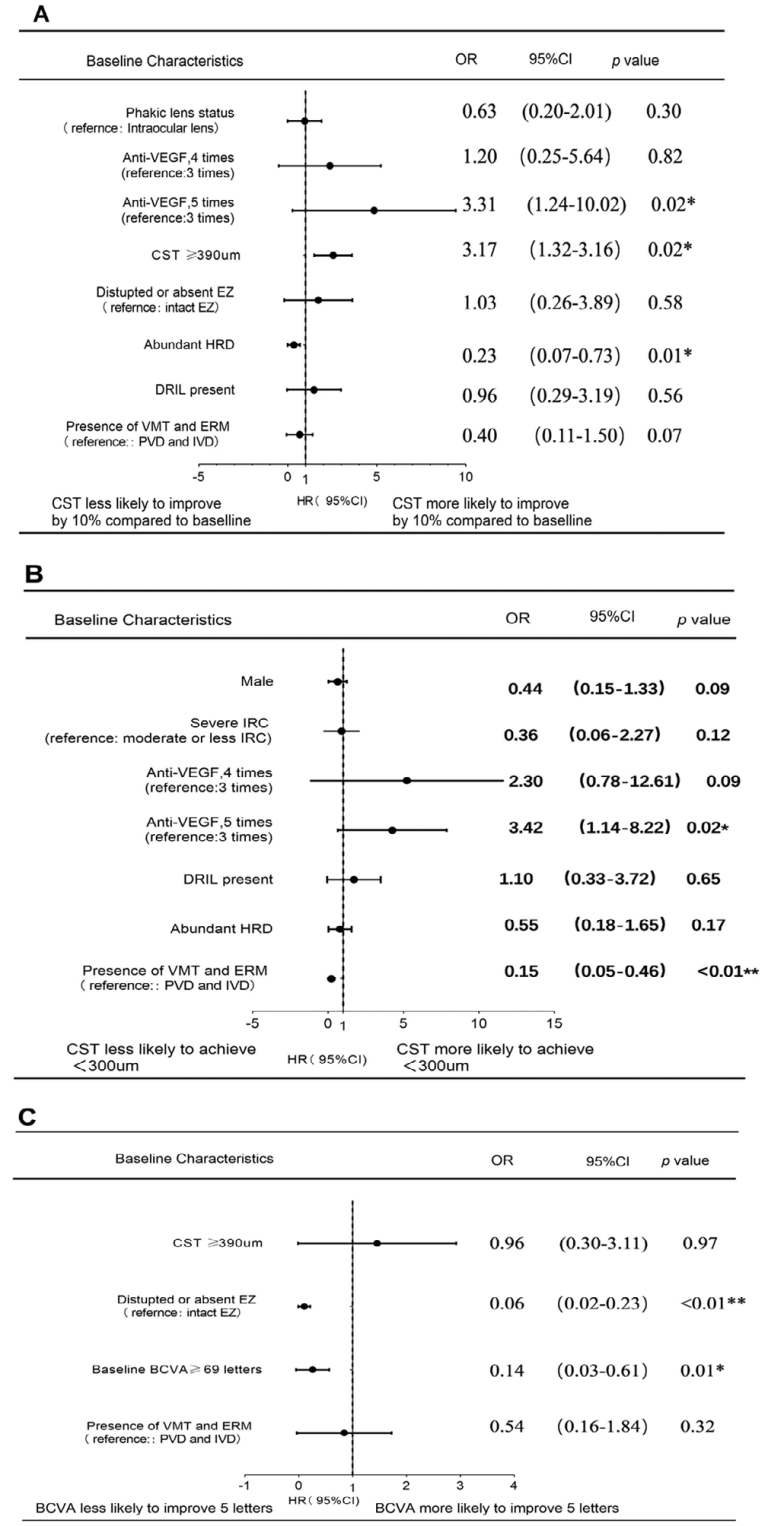
Multivariate analysis of imaging biomarkers and outcomes after anti-VEGF therapy in patients with diabetic macular edema (DME). (A) Endpoint 1: CST reduction of 10% or more from baseline. (B) Endpoint 2: CST < 300 μm at 6 months. (C) Endpoint 3: BCVA improvement of five letters or more from baseline. *P <0.05; *P<0.01.

Multivariate analysis of biomarkers related to the efficacy of anti-VEGF therapy: Further multivariate logistic regression analysis was performed on factors in univariate analysis that may be relevant to the 6-month outcome. The results revealed that five injections (*OR* 3.31; 95%*CI* 1.24–10.02; *P* = 0.02) and baseline CST ≥ 390 μm (*OR* 3.17; 95%*CI* 1.32 ~ 3.16; *P* = 0.02) were related to the decrease of CST ≥ 10%; abundant HRD(*OR* 0.23; 95%*CI* 0.07–0.73; *P* = 0.01) had negative effect on the decreased CST ≥ 10% (Fig. 2A). Eyes that received five injections within 6 months (*OR* 3.42; 95% *CI* 1.14–8.22; *P* = 0.02) were more likely to reach the end point of CST < 300 μm; Eyes with VMT or ERM presence at baseline (*OR* 0.15; 95% *CI* 0.05–0.46; *P* < 0.01) were less likely to reach the endpoint of CST < 300 μm (Fig. 2B). Negative predictors of BCVA improvement by more than five letters included baseline BCVA ≥ 69 letters (*OR* 0.14; 95% *CI* 0.03–0.61; *P* = 0.01) and complete or partial destruction of EZ (*OR* 0.06; 95% *CI* 0.02–0.23; *P* < 0.01)(Fig. 2C). Although severe DME staging was an important factor in univariate analysis, it was not included in multivariate analysis because it was composed of other factors included in the model.

**Fig. 3:**
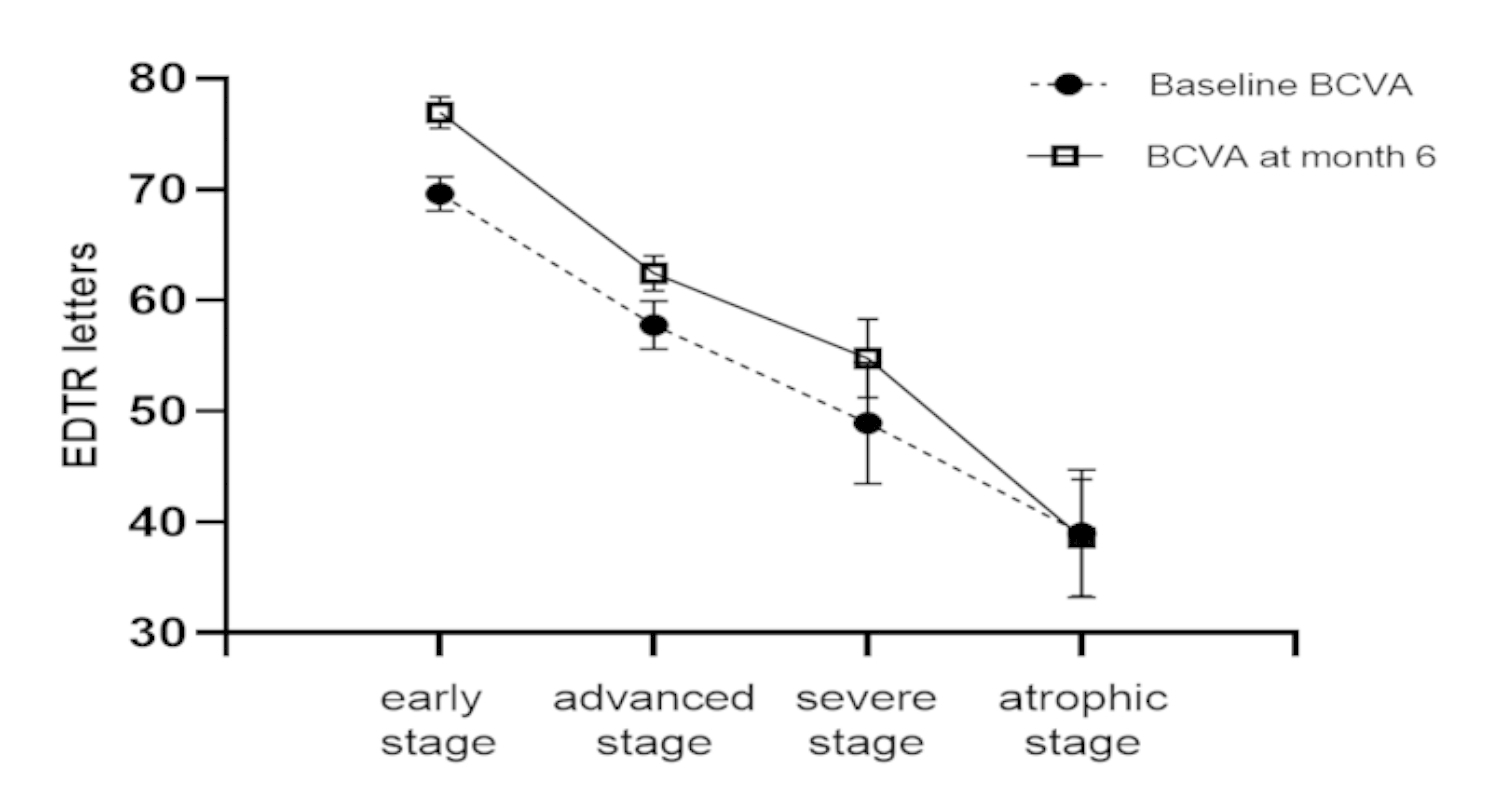
Mean BCVA EDTR letters in different DME stages before and after 6 months of treatment of anti-VEGF

**Fig. 4:**
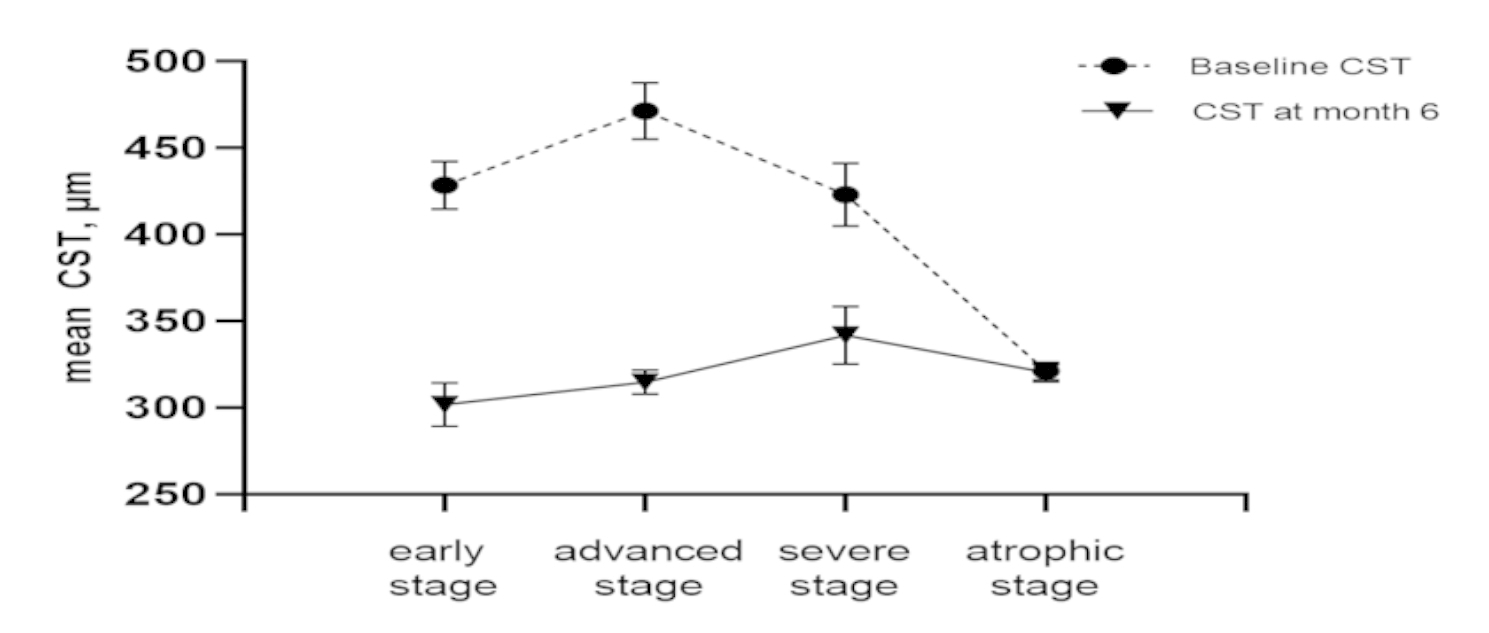
Mean CST in different DME stages before and after 6 months of treatment of anti-VEGF

Prediction and analysis of anti-VEGF effect through TCED-HFV grading: TCED-HFV grade was negatively correlated with BCVA at baseline and BCVA at 6 months (Kendall’s tau-b=-0.39 and − 0.55, *P* < 0.01). At 6 months of follow-up, there was no significant difference between the four grades on changing letters. (Kruskal-Wallis test result: H = 2.48 *P* = 0.48) (Fig. 3). TCED-HFV grade was positively correlated with CST at 6 months (Kendall’s tau-b = 0.19, *P* = 0.049); There was an inverse correlation with the reduction in CST during follow-up (Kendall’s tau-b=-0.32, *P* < 0.01)(Fig. 4).

**Fig. 5:**
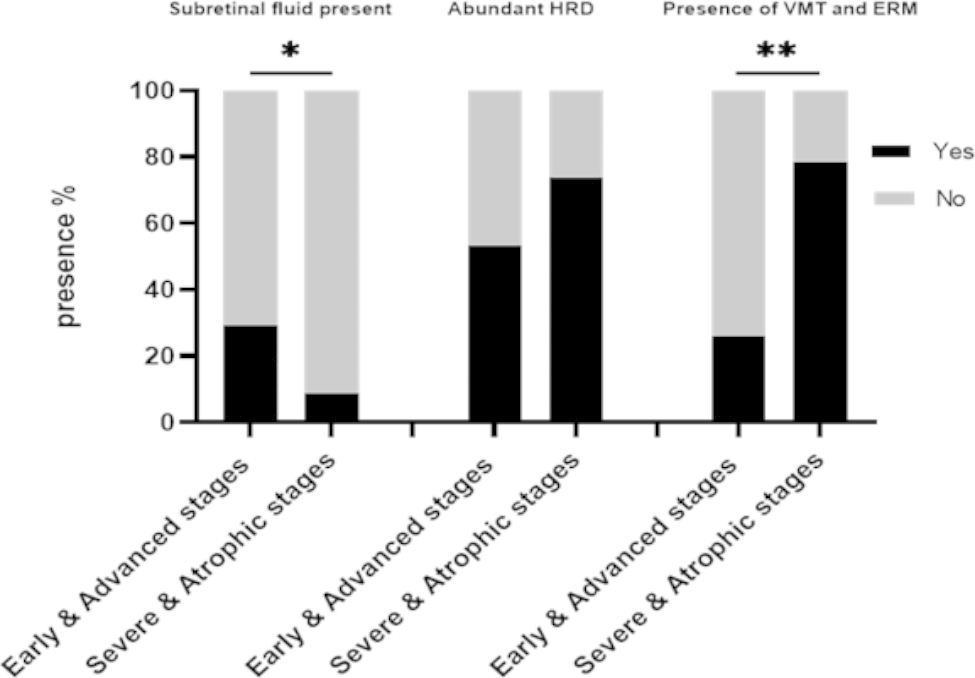
Imaging characteristics in different DME stages

Early & advanced DME was more likely to have SRF(*χ*^*2*^ = 3.90 *P* = 0.048); Severe & atrophic DME was more often combined with VMT or ERM (χ2 = 28.57, *P* < 0.01) (Fig. 5).

## Discussion

Previous studies have demonstrated the efficacy of anti-VEGF on DME. The results of the current study revealed that 55.6% of treated eyes achieved a BCVA gain of greater than or equal to five letters, whereas 29.6% maintained stable vision. However, 14.8% of the eyes exhibited a decrease in BCVA of more than five letters. As in previous studies [7, 12, 13], our findings confirm a difference in the response of affected eyes to anti-VEGF treatment. We observed a significant correlation between the number of injections and the anatomical results. However, after adjusting the number of injections in multivariate analysis, we found that other biomarkers were significantly correlated with the results. By evaluating the potential predictors and establishing a prognostic model for DME, ophthalmologists can personalize the management of DME based on the risk of vision loss.

some biomarkers have been described as associated with prognosis, but it is difficult to explain the predictive effect of any single factor on anatomical and functional prognosis after treatment because DME is a complex multifactorial disease. Baseline VA is the most concerned marker of visual function prognosis. However, there are some issues. First, the ceiling effect of eyes with good baseline vision means that changes in VA are not a good indicator of the effectiveness of treatment in this group because the treatment of these eyes is designed to prevent vision loss. A post-hoc analysis of DRCR protocol I (361 eyes) revealed that patients with a lower score (≤ 65 letters) had more improvement in BCVA than those with DME with a higher baseline letter score [14]。 Santos’s study found that patients with lower baseline letter scores (< 49 letters) had a significant improvement in BCVA (+ 9.4 letters) at the end of the 6-month follow-up compared with DME patients with higher baseline letter scores (+ 3.2 letters)[15]. Our study also demonstrated that baseline BCVA ≥ 69 letters was a negative predictor of endpoint visual acuity improvement of more than five letters (*OR* 0.14;95%*CI* 0.03–0.61; *P* = 0.01). Although much emphasis is placed on the initial VA, these observations suggest that structural changes precede the decline of visual function. Therefore, it is necessary to study further the relationship between the detailed characteristics of imaging and the prognosis. Many DME-related biomarkers based on SD-OCT imaging, such as CST, choroidal thickness, HRD, HE, SRF, IRC, EZ or ELM rupture, and DRIL, have been proven to be general prognostic biomarkers of anatomical or functional outcomes [16–20]. In the future, the information parameters of macular perfusion provided by OCTA will be further used in the evaluation system [21]. A large amount of HRD predicts a slight reduction in CST after anti-VEGF therapy. Eyes present with VMT or ERM at baseline are less likely to reach the endpoint of CST < 300 μm. However, the decrease in CST does not reflect the final VA results, nor is it a clear predictor of VA [12, 22, 23]. An analysis of the RIDE and RISE trials revealed that patients with no significant changes in fovea thickness after treatment with anti-VEGF had similar visual acuity and DR improvements to those with immediate retinal thinning [24]. These findings suggest that although CST can reflect the amount of liquid in DME, it is not a clear visual prognostic indicator. CST and its fluctuation is only modestly correlated with vision in DME [25]. CST with similar thickness may have different neuronal atrophy and Müller cell dysfunction, which may be more related to visual results than CST [26]. Our study also confirmed the existence of baseline HRD as a predictor of poor DME response to anti-VEGF therapy. Disrupted or absent EZ was a negative predictor of BCVA gain of greater than or equal to five letters. The quantification of CST must be combined with other morphological features to predict visual outcomes.

Compared with a single factor, the comprehensive score system of the combination of multiple factors may predict the prognosis of anti-VEGF treatment of DME from anatomical and functional dimensions. We cited the TCED-HFV grading system to verify that this grading can accurately reflect the severity of DME and provide information for prognosis. BCVA decreased at baseline with an increased grade of more severe DME, and 6 months after treatment, the heavier the grade associated with poor visual acuity; CST increased with a more severe DME grade after anti-VEGF therapy; The reduction in CST during follow-up decreased with a more severe DME grade. The grading system of DME also allows us to understand the pathophysiological process of DME. Previous studies have suggested that SRF at baseline was associated with visual and anatomical benefits after anti-VEGF and that SRF had been considered a protective factor [27, 28]. The study by Serra et al. suggested that serous macular detachment is mainly seen in early DME [28]. Our study found that compared with severe and atrophic DME, SRF was more likely to exist in the early and advanced group(*χ2* = 3.90, *P* = 0.048). In the future, larger samples and longer time dimensions are needed to explore the relationship between SRF and disease course and disease severity. [29]It is well known that DME patients have a high incidence of vitreomacular interface abnormality (VMIA). ERM and VMT occur in patients with incomplete posterior vitreous detachment (IVD). In our study, IVD accounted for 35.8%, VMT 19.8%, and ERM 24.7%. Moreover, we found that severe and atrophic DME was more often combined with VMT or ERM (*χ2* = 28.57, *P* < 0.01). The pathogenesis of DME-related ERM is different from that of idiopathic ERM and is thought to be related to chronic inflammation caused by hypoxia, oxidative stress, and up-regulation of VEGF [30].

The limitation of this study is that it is a single-center retrospective study with a relatively small number of cases. Our cohort included patients who received more than three consecutive anti-VEGF treatments and followed up for more than 6 months, excluding patients who underwent vitrectomy before and during follow-up, which may be affected by selection bias. HRD and DRIL were manual measurements, and there might be measurement errors. However, this study is based on a real-world study, our results may have broad applicability. Patients with a history of vitrectomy were excluded from this study because different medications may have different bioavailability and because surgery may affect the qualitative and quantitative analysis of OCT. To try to avoid judgment errors, two masked, independent, experienced raters were used in this study.

## Conclusion

This study verified the predictive value of the TCED-HFV grading system in anti-VEGF in the treatment of DME in real clinical scenarios. In clinical work, the system is convenient for comprehensive evaluation of the affected eyes and is a useful tool and prediction model. This study explored the OCT biomarkers related to the anatomical targets and visual effects of anti-vascular endothelial growth factor therapy. This study also proved that although individual factors may not be sufficient, the weighted combination of the relative importance of these factors may form a model to predict prognosis.

## Electronic supplementary material

Below is the link to the electronic supplementary material.

Supplementary Material 1

## Data Availability

All data needed to evaluate the conclusions in the paper are present in the paper and/or the Supplementary Materials.

## Abbreviations

DME: Diabetic macular edema
anti-VEGF: Anti-vascular endothelial growth factor
PRP: Panretinal photocoagulation
NPDR: Nonproliferative diabetic retinopathy
PDR: Proliferative diabetic retinopathy
BCVA: Best-corrected visual acuity
ETDRS: Early Treatment Diabetic Retinopathy Study
CST: Central subfield thickness
IRC: Intraretinal cyst
EZ: Ellipsoid zone
ELM: External limiting membrane
DRIL: Disorganized retinal inner layers
HRD: Hyperreflective dot, HE hard exudates
ERM: Epiretinal membrane
VMT: Vitreomacular traction
PDV: Complete posterior vitreous detachment
IDV: Incomplete posterior vitreous detachment
OR: Odds ratio
CI: Confidence interval
